# Subjective versus objective refraction in healthy young adults

**DOI:** 10.1186/s12886-024-03340-w

**Published:** 2024-02-20

**Authors:** Yuval Kozlov, Michael Kinori, Sharon Armarnik, Tal Yahalomi, Aya Ekshtein, Leora Levian, Daphna Mezad-Koursh, Joseph Pikkel, Oded Ben-Ari

**Affiliations:** 1 Department of Military Medicine, Faculty of Medicine, The Hebrew University of Jerusalem, Jerusalem, Israel; 2 The Israeli Air Force Aeromedical Center, Tel Hashomer, Israel; 3 Department of Ophthalmology, Assuta Ashdod Medical Center, Ashdod, Israel; 4 Faculty of Health Sciences, Ben-Gurion University of the Negev, Beer-Sheva, Israel; 5 The Goldschleger Eye Institute, Sheba Medical Center, Tel Hashomer, Israel; 6 Department of Ophthalmology, Tel Aviv Medical Center, Tel Aviv, Israel; 7 The Adelson School of Medicine, Ariel University, Ariel, Israel

**Keywords:** Astigmatism, Autorefraction, Hyperopia, Myopia, Subjective refraction, Visual acuity

## Abstract

**Purpose:**

To evaluate objective and subjective refraction differences in healthy young adults.

**Methods:**

Data concerning candidates for the Israeli Air Force Flight Academy, as well as active air force pilots in all stages of service who underwent a routine health checkup between the years 2018 and 2019 were retrospectively analyzed. Objective refraction measured using a single autorefractometer was compared with subjective refraction measured by an experienced military optometrist during the same visit. The results were converted to power vectors (spherical equivalent [SE], J0, and J45). To interpret astigmatism using power vector values, the cylinder power (Cp) was determined.

**Results:**

This study included 1,395 young adult participants. The average age was 22.17 years (range, 17–39, 84.8% males). The average SE was − 0.65 ± 1.19 diopter (D) compared with − 0.71 ± 0.91D in the auto- and subjective refraction, respectively (*p* = 0.001). Cp was 0.91 ± 0.52D and 0.67 ± 0.40D, respectively (*p* < 0.001). This difference was more common in older participants (*p* < 0.001). J0 and J45 value differences were not significant. The absolute SE value of subjective refraction was lower in the myopic (*p* < 0.001) and hyperopic (*p* < 0.001) patients.

**Conclusions:**

Young hyperopic participants tended to prefer “less plus” in subjective refraction compared with autorefraction. Young myopic participants tended to prefer “less minus” in subjective refraction compared with autorefraction. All participants, but mainly older participants, preferred slightly “less Cp” than that measured using autorefraction; The astigmatic axis did not differ significantly between the methods.

## Introduction

Refractive error measurement is a crucial component of patient management decisions made by ophthalmologists and optometrists. Because visual perception depends not only on optical factors but also on the neural force, and since patients are most likely to accept their preferred values for their spectacle prescriptions, subjective refraction is considered the gold standard for refractive error assessment and spectacle prescription in cooperative patients [1, 2].

Despite subjective refraction being considered highly reliable, it can be altered by the intra-examiner (same examiner) and the inter-examiner (different examiners) and is reported to be within 0.25 to 0.50D [3]. In addition, it is necessary to decrease the examination time, making it more accessible to less-trained optometrists while ensuring quality and accuracy. Finally, in small children and non-cooperative adults, subjective refraction may not be reliable.

Since the 1970s, objective refraction using autorefractometers has gained popularity for clinical, screening, and research purposes owing to its relatively high accuracy, repeatability, and ease of use by laypeople [4–7]. The ideal standard for diagnosing the objective refractive errors in both children and adults up to the age of 50 involves the use of cycloplegic (“wet”) refraction [8]; however, in most settings, automated refractors are used without the use of cycloplegic drugs (“dry refraction”). Most autorefractors use built-in automatic fogging mechanisms to avoid accommodation during measurement [9] and noncycloplegic autorefraction has reasonable accuracy and repeatability. Nevertheless, in conventional autorefractors accommodation may not be completely neutralized, resulting in the measurement being more myopic (pseudomyopia) [10]. Therefore, inaccurate measurements using autorefractometers are primarily due to accommodation resulting from inadequate autofogging mechanisms [11, 12]. This overcorrection of myopes and undercorrection of hyperopes is especially pertinent in children without cycloplegia who have high accommodative reserve [9, 13–15]. Moreover, objective methods may not appropriately consider higher-order aberrations that can influence visual acuity [16]. These aberrations are also altered by pupil size [16]. Therefore, autorefraction is valuable but serves only as a starting point for the subjective refraction procedure [3, 11].

This study endeavors to assess the reliance on autorefractometers within a cooperative and educated demographic of young adults, specifically Israeli Air Force (IAF) pilots and flight academy candidates. In this population, the precise evaluation of refractive error is not only crucial but also reflective of their occupational demands. By examining the disparities between subjective and objective refraction, we aim to gauge the suitability of autorefraction in adults capable of reliable self-reporting, especially those with high stakes in the accuracy of their visual assessment.

In addition, the impracticality of conducting subjective refraction in non-cooperative adults underscores the necessity for alternative approaches. Within this distinct population, we extrapolate conclusions regarding the correct refractive correction from a cooperative young adult cohort, as outlined in this study. Our research imparts valuable insights into this clinical scenario, furnishing practical guidelines derived from a substantial patient cohort.

## Methods

This study was approved by the local ethics committee of the Israeli Defense Forces (IDF) and involved a retrospective database analysis. Medical data were extracted from the Israeli Air Force Aeromedical Center’s (IAFAC) archive. Medical records of candidates for the Israeli Air Force Flight Academy, including soldiers currently training in the academy as well as active military pilots who were examined between the years 2018–2019 were included. Candidates for the Israeli Air Force Flight Academy underwent several medical tests, including meticulous eye examinations. Active military pilots undergo annual eye examinations. All examinations were performed by a well-trained military optometrist and ophthalmologist in a room with standard illumination. Each examination included automatic autorefraction and subjective refraction. Noncycloplegic autorefraction and subjective refraction were performed using a Tomey RT-7000 Autorefractometer (Nagoya, Japan, 2017). An experienced military optometrist conducted Subjective Refraction utilizing a standard phoropter. Patients were instructed to focus on the Snellen Chart while a range of lenses was presented to them. The optometrist subsequently fine-tuned the power of the lenses in the trial frames, guided by the patients’ subjective feedback regarding improvements in their vision.

For statistical purposes, only the right eye (RE) was analyzed, and only subjects under 40 years old were included in the study analysis. None of the study subjects had a history of ocular comorbidities.

For statistical analysis of the comparison between the objective and subjective refraction measurements, the spherical equivalent (SE) and power vectors were derived using the equations developed by Miller [17] for Fourier power vector analysis [18]. The SE was calculated from the sphere (S) and the cylinder (C) using the following equation:

$$SE=S+\frac{C}{2}$$

The rectangular vectors J0 and J45 were then calculated using the cylinder (C) and axis (A) in the following equations:

$$\begin{array}{c}\alpha=A\;X\;2\;\frac\pi{180}\\J_0=-\frac C2X\;Cos(2\;\boldsymbol\alpha\boldsymbol)\\J_{45}=-\frac C2X\;Sin(2\;\boldsymbol\alpha\boldsymbol)\end{array}$$

J0 is the Jackson cross-cylinder power along the 90° and 180° axes. J45 is the Jackson cross-cylinder power along the 45° and 135° axes. *α* is the axis of the flat meridian. To interpret the astigmatism using power vector values, the following equation [19] was used to determine the cylindrical power (Cp):

$$Cp= 2\sqrt{{J0}^{2}+{J45}^{2}}$$

For statistical analysis, using an autorefractometer measurement, a participant was considered to be myopic if his right eye (RE) SE was ≤-0.50 dpt (D). If the RE SE was ≥ + 0.5D, the participant was considered to be hyperopic. A participant with an RE SE between − 0.5D and + 0.5D was considered to be emmetropic. After deriving the Cp from the power vectors, astigmatism was defined as ≥ 0.75D [20].

### Data analysis

Data were analyzed using IBM SPSS Statistics software version 25. Descriptive statistics are presented using means and standard deviations for continuous variables and frequencies and percentages for discrete variables. To assess whether J0, J45, cylinder, or SE differed between the measurements, paired *t*-tests were performed.

To determine how different factors may contribute to a more significant difference between the measurements, two-sample *t*-tests were performed on the mean difference of each spherical cylinder or SE in relation to astigmatism, myopia, hyperopia and age.

Chi-square and Fisher’s exact tests were used to evaluate whether these factors influenced the prevalence of clinically significant changes between the two measurements. Clinically significant measurements were determined as a change of at least 0.75D for the sphere, cylinder, or SE.

## Results

A total of 1,395 young adults participated in this study. The average age was 22.17 (range, 17–39) years. Most participants were males (1,184, 84.8%), and 682 (48.8%) participants were classified as having myopia, with a median SE of -1.12D (range, -0.50–-8.38 D). Another 145 (10.4%) were hyperopic, with a median SE of +0.75 D (range, +0.50–+5.12D). Astigmatism was identified in 971 (69.6%) participants (median 1.00 D, range 1 to 4.50D).

The mean SE using autorefraction was − 0.65 ± 1.19D compared to -0.71 ± 0.91D in subjective refraction (*p* = 0.001). The mean Cp using autorefraction was 0.91 ± 0.52D compared with 0.67 ± 0.40D in subjective refraction (*p* < 0.001). However, the differences between J0 and J45 were not significant. Inter-measurement comparisons of the mean differences between auto- and subjective refractions are presented in Table 1.

**Table 1:** An inter-measurement comparison of mean differences between auto- and subjective refraction

Variable	AR	SR	Mean Difference	*P* value
**SE**	-0.65 ± 1.19	-0.71 ± 0.91	0.05	***p*** ** = 0.001**
**J0**	0.01 ± 0.47	0.01 ± 0.35	-0.00	*p* = 0.86
**J45**	-0.04 ± 0.22	-0.03 ± 0.17	-0.00	*p* = 0.25
**Cp**	0.91 ± 0.52	0.67 ± 0.40	0.23	***p*** ** < 0.001**

*SE* Spherical Equivalent, *Cp* Cylindrical power, *AR* Autorefraction, *SR* Subjective refraction

Subgroup analysis of clinically significant changes indicated that the difference in SE between both measurements in participants who were classified as hyperopic was significant (*p* < 0.001). In this group, subjective SE was lower than SE as measured using the autorefractometer. In participants with myopia, the subjective SE was also lower (in absolute value) than SE measured using the autorefractometer (*p* < 0.001). Subgroup pairwise comparison for SE differences is furtherly presented in Table 2.

**Table 2:** The influence of contributing factors on the spherical equivalent differences between autorefraction and subjective refraction

Variable	SE AR	SE SR	Mean difference	*P* value
**Myopia**				
Yes	-1.47 ± 1.15	-1.18 ± 1.09	-0.29 ± 0.58	***p*** ** < 0.001**
No	0.12 ± 0.51	-0.25 ± 0.30	0.38 ± 0.40	
**Hyperopia**				
Yes	0.87 ± 0.60	-0.04 ± 0.54	0.91 ± 0.47	***p*** ** < 0.001**
No	-0.83 ± 1.12	-0.78 ± 0.92	-0.04 ± 0.53	
**Age**				
< 21 years	-0.67 ± 1.27	-0.73 ± 0.99	0.06 ± 0.65	*p* = 0.32
≥ 21 years	-0.63 ± 1.04	-0.67 ± 0.78	0.03 ± 0.52	

*SE* Spherical Equivalent, *AR* Autorefraction, *SR* Subjective refraction

In participants classified as having astigmatism (≥ 0.75D), Cp was higher when measured using the autorefractor than when measured using subjective refraction (*p* < 0.001). Hyperopic refraction did not alter this result (*p* = 0.50), while myopic refraction had, as myopic subjects had higher Cp when measured using the autorefractor (*p* = 0.03). The differences between J0 and J45 remained insignificant in all the groups.

The SE between measurements was not different in older (21 years or older) versus younger patients (*p* = 0.32). However, the difference in Cp values was significant in the older group, with subjective refraction showing a lower Cp value (0.32D in the older group vs., 0.19D in the younger group) than autorefractometry value (*p* < 0.001).

## Discussion

In this study, we examined a large cohort of healthy young adults without any known ocular pathologies We aimed to examine and measure the differences between objective and subjective refractions.

Refraction is likely the most frequent measurement in clinical practice. However, subjective adjustment is necessary to determine the final refraction. Reducing the time spent in refraction is an appropriate method for increasing clinical efficacy and may allow clinicians to perform deeper eye examinations. It is crucial to consider that even a 0.25D difference in prescriptions is clinically significant and may impact an individual’s quality of life [3]. In this large study cohort, our findings indicated that autorefractors were satisfactory for a preliminary refraction but were not sufficient as substitutes for conventional subjective refraction, as also reported by Goss et al. [3]. If glasses are to be prescribed based on noncycloplegic autorefractor readings, it is necessary to obtain a number close to the subjective refraction, especially in a noncooperative adult patients or in countries with a lack of qualified optometrists and ophthalmologists.

Our cohort comprised mainly young adults with a mean age of 22.17 years. The SE measured by subjective refraction was only slightly more myopic than that found using the autorefractometer. In the subgroup analyses, the absolute value of the subjective SE was lower in both myopes and hyperopes, IE less myopic in the myopic group and less hyperopic in the hyperopic group. This may be because myopes tend accommodate more during the autorefraction measurement compared to subjective refraction measurement. In contrast, in the participants with hyperopia, there might be lesser accommodation during autorefraction compared to subjective refraction or overestimation of hyperopia by the autorefractometer. An underestimation of hyperopia (or overestimation of myopia) due to instrument myopia has been described but studies included a relatively low number of participants and/or, most importantly, myopes and hyperopes were not discussed separately [21–23].

In hyperopes - less hyperopia was found during subjective refraction. This could be explained by an increased accommodation during subjective refraction -perhaps in order to concentrate rigorously and read the 20/20 line without errors in front of the optometrist during an important eye exam. This could be especially true if hyperopia is not corrected, which leads to a constant accommodative effort in the daily life of young hyperopic adults, which increases even more during the exam. In contrast, facing an autorefractometer, where concentration is not needed, the participant, who is unaware of the test result, may be more relaxed.

On the other hand, a young, low myopic adult’s accommodation might not be that strong, particularly if left uncorrected. Furthermore, adults with myopia are more likely to have undergone multiple eye examinations prior participating in our study, and/or know that they need optical correction for better vision and therefore are less stressful about the eye exam. In contrast, young participants with hyperopia who may not have had an eye examination during their adult life and are not aware of any refractive error may need to increase accommodation in a stressful environment of a crucial sight testing.

The astigmatic error in the subjective refraction was found to be lower than that in the autorefraction measurement. This gap in astigmatic power was greater in the older and myopic participants. This result indicated that especially older myopic participants preferred less Cp. The cylindrical vector did not differ between measurements, as was previously reported by Bullimore et al. [11]. In contrast, Jorge et al. showed that the autorefractor provided more positive values than the subjective refraction for the J0 vector, whereas the J45 component was more negative for the autorefractor [21].

Our findings may not fully represent visual performance in the general population, as we only included young healthy adults without any known ocular issues with a very high motivation to excel in vision testing. In a previous study, we described this specific group as having a very high visual performance [20]. On the other hand, our large cohort of very motivated healthy participants provides high-quality and reliable results that shed light on subjective refraction compared with automated refraction in different refractive statuses and ages.

In conclusion, based on our results, the expected subjective refraction based on autorefractometer results in young adults will be “less” hyperopic in hyperopes and “less myopic” in myopes. The Cp will also be reduced by about 0.25D in those with myopia and hyperopia, and mainly in older patients.

## Data Availability

To request access to the raw data, kindly reach out to Dr. Michael Kinori via email at michaelkinori@gmail.com. Please be aware that any publication of raw data will necessitate military approval.

## References

[1] Carracedo G, Carpena-Torres C, Serramito M, Batres-Valderas L, Gonzalez-Bergaz A (2018). Comparison between aberrometry-based binocular refraction and subjective refraction. Transl Vis Sci Technol.

[2] Bamdad S, Momeni-Moghaddam H, Abdolahian M, Pinero DP (2022). Agreement of wavefront-based refraction, dry and cycloplegic autorefraction with subjective refraction. J Optom.

[3] Goss DA, Grosvenor T (1996). Reliability of refraction--a literature review. J Am Optom Assoc.

[4] Allen PM, Radhakrishnan H, O’Leary DJ (2003). Repeatability and validity of the PowerRefractor and the Nidek AR600-A in an adult population with healthy eyes. Optom Vis Sci.

[5] Cleary G, Spalton DJ, Patel PM, Lin PF, Marshall J (2009). Diagnostic accuracy and variability of autorefraction by the tracey visual function analyzer and the Shin-Nippon NVision-K 5001 in relation to subjective refraction. Ophthalmic Physiol Opt.

[6] Salmon TO, West RW, Gasser W, Kenmore T (2003). Measurement of refractive errors in young myopes using the COAS Shack-Hartmann aberrometer. Optom Vis Sci.

[7] Yeung IY, Mantry S, Cunliffe IA, Benson MT, Shah S (2004). Correlation of Nidek OPD-Scan objective refraction with subjective refraction. J Refract Surg.

[8] Morgan IG, Iribarren R, Fotouhi A, Grzybowski A (2015). Cycloplegic refraction is the gold standard for epidemiological studies. Acta Ophthalmol.

[9] Choong YF, Chen AH, Goh PP (2006). A comparison of autorefraction and subjective refraction with and without cycloplegia in primary school children. Am J Ophthalmol.

[10] Guo R, Shi L, Xu K, Hong D (2022). Clinical evaluation of autorefraction and subjective refraction with and without cycloplegia in Chinese school-aged children: a cross-sectional study. Transl Pediatr.

[11] Bullimore MA, Fusaro RE, Adams CW (1998). The repeatability of automated and clinician refraction. Optom Vis Sci.

[12] Mallen EA, Wolffsohn JS, Gilmartin B, Tsujimura S (2001). Clinical evaluation of the Shin-Nippon SRW-5000 autorefractor in adults. Ophthalmic Physiol Opt.

[13] Sankaridurg P, He X, Naduvilath T, Lv M, Ho A, Smith E, Erickson P, Zhu J, Zou H, Xu X (2017). Comparison of noncycloplegic and cycloplegic autorefraction in categorizing refractive error data in children. Acta Ophthalmol.

[14] Sun YY, Wei SF, Li SM, Hu JP, Yang XH, Cao K, Lin CX, Du JL, Guo JY, Li H, et al. Cycloplegic refraction by 1% cyclopentolate in young adults: is it the gold standard? The Anyang University Students Eye Study (AUSES). Br J Ophthalmol. 2019;103(5):654–8. 10.1136/bjophthalmol-2018-312199 29930099

[15] Zhao J, Mao J, Luo R, Li F, Pokharel GP, Ellwein LB (2004). Accuracy of noncycloplegic autorefraction in school-age children in China. Optom Vis Sci.

[16] Guirao A, Williams DR (2003). A method to predict refractive errors from wave aberration data. Optom Vis Sci.

[17] Miller JM (2009). Clinical applications of power vectors. Optom Vis Sci.

[18] Thibos LN, Wheeler W, Horner D (1997). Power vectors: an application of Fourier analysis to the description and statistical analysis of refractive error. Optom Vis Sci.

[19] Liu YC, Chou P, Wojciechowski R, Lin PY, Liu CJ, Chen SJ, Liu JH, Hsu WM, Cheng CY (2011). Power vector analysis of refractive, corneal, and internal astigmatism in an elderly Chinese population: the Shihpai eye study. Invest Ophthalmol Vis Sci.

[20] Armarnik S, Kozlov Y, Yahalomi T, Ekshtein A, Levian L, Gurfinkel Y, Tehori O, Ben-Ari O, Kinori M (2022). The influence of refractive state and heterophorias on visual acuity and stereoacuity in healthy young adults. J AAPOS.

[21] Jorge J, Queiros A, Almeida JB, Parafita MA (2005). Retinoscopy/autorefraction: which is the best starting point for a noncycloplegic refraction?. Optom Vis Sci.

[22] Nayak BK, Ghose S, Singh JP (1987). A comparison of cycloplegic and manifest refractions on the NR-1000F (an objective auto refractometer). Br J Ophthalmol.

[23] Zadnik K, Mutti DO, Adams AJ (1992). The repeatability of measurement of the ocular components. Invest Ophthalmol Vis Sci.

